# Unresolved Issues for Utilization of Atypical Antipsychotics in Schizophrenia: Antipsychotic Polypharmacy and Metabolic Syndrome

**DOI:** 10.3390/ijms18102174

**Published:** 2017-10-18

**Authors:** Sang Won Jeon, Yong-Ku Kim

**Affiliations:** 1 Department of Psychiatry, Kangbuk Samsung Hospital, Sungkyunkwan University School of Medicine, Seoul 03181, Korea; sangwonyda@hanmail.net; 2 Department of Psychiatry, Korea University Ansan Hospital, Korea University College of Medicine, Ansan 15355, Korea

**Keywords:** atypical antipsychotics, schizophrenia, polypharmacy, treatment resistance, metabolic syndrome

## Abstract

Atypical antipsychotics (AAP) are the prevailing form of schizophrenia treatment today due to their low side effects and superior efficacy. Nevertheless, some issues still need to be addressed. First, there are still a large number of patients with treatment-resistant schizophrenia (TRS), which has led to a growing trend to resort to AAP polypharmacy with few side effects. Most clinical treatment guidelines recommend clozapine monotherapy in TRS, but around one third of schizophrenic patients fail to respond to clozapine. For these patients, with clozapine-resistant schizophrenia AAP polypharmacy is a common strategy with a continually growing evidence base. Second, AAP generally have great risks for developing metabolic syndrome, such as weight gain, abnormality in glucose, and lipid metabolism. These metabolic side effects have become huge stumbling blocks in today’s schizophrenia treatment that aims to improve patients’ quality of life as well as symptoms. The exact reasons why this particular syndrome occurs in patients treated with AAP is as yet unclear though factors such as interaction of AAP with neurotransmitter receptors, genetic pholymorphisms, type of AAPs, length of AAP use, and life style of schizophrenic patients that may contribute to its development. The present article aimed to review the evidence underlying these key issues and provide the most reasonable interpretations to expand the overall scope of antipsychotics usage.

## 1. Introduction

The advent of atypical antipsychotics (AAP) has enabled clinicians to treat schizophrenia with an increased efficacy and fewer side effects when compared to typical antipsychotics (TAP) [1]. AAP show a minimal or absent propensity to induce extrapyramidal symptoms at therapeutic dosage, a lower risk to induce hyperprolactinemia, and a higher efficacy in managing both positive and negative symptoms in schizophrenic patients. Nevertheless, a few challenges remain to be overcome.

For one, there are still patients with treatment-resistant schizophrenia (TRS), which has led to a growing trend to resort to AAP polypharmacy, with few side effects [2,3]. This has created a gap between the treatment guidelines and actual clinical practice, as most clinical treatment guidelines recommend monotherapy in patients with TRS. The reasons for the antipsychotic monotherapy may be associated with the fact that TAPs were assumed to have similar mechanism of action and that combination use of more than two TAPs would offer no benefit over that of monotherapy with the agents. However, because the newer AAPs have the notable features in their diverse pharmacologic action and lower adverse event profiles, many clinicians have an interest in using the AAP combination therapy. Up to the present date, we are in lack of the evidence of the rationale and background of this practice.

Second, AAP generally have great risks for developing metabolic syndrome, such as weight gain, abnormality in glucose, and lipid metabolism [4]. The mechanisms of AAP-induced metabolic syndrome are variable and complex, but much clinical research has shown that AAP may affect body weight through two prime mechanisms. Increased appetite is probably related to the interaction of AAP with neuronal receptors to dopamine, serotonin, and histamine. Additional metabolic disruption may be related to the effects of AAP-induced insulin sensitivity. Patients with schizophrenia are not only at risk of diabetes mellitus (DM), but also taking AAP further increases the chance of developing non-insulin-dependent hyperglycemia. These metabolic adverse effects are often associated with non-compliance and medical problems.

Third, it is unclear whether AAP may directly decrease suicidality in schizophrenic patients. Clozapine is the only AAP that gathered evidence as an effective treatment for reducing suicidality in schizophrenia. However, there is still a lack of research to determine as to whether clozapine can directly reduce suicide because of its antidepressant effect, or whether other pharmacologic factors might be involved.

The aim of this study was to review the evidence supporting the discussion points for the two key antipsychotics issues—polypharmacy and metabolic disorder—and to provide the most reasonable interpretations so as to broaden the overall scope of atypical antipsychotics usage. These two topics are not new, but are so important in the treatment of schizophrenia, that integrated and organized knowledge must be constantly updated.

## 2. Methods

The source of literature was the electronic database MEDLINE via PubMed (1990–2017). The initial search strategy included the use of a combination of the following thesaurus terms: [atypical antipsychotics/second generation antipsychotics] AND [polypharmacy/combination/metabolic syndrome]. The inclusion criteria included: (i) original articles examining atypical antipsychotics mechanisms in the human subjects; (ii) “Practice Guidelines” for the “Type of Article” was searched from the “Systemic Review” published by PubMed; (iii) articles written in English. The exclusion criteria included: (i) letters to editors and editorials without data; and, (ii) studies outside the time window. Based on the inclusion and exclusion criteria, we reviewed the titles of all of the citations and retrieved relevant abstracts for more detailed evaluation. When there was uncertainty, we studied the full article. We also hand-searched the reference list of relevant studies and reviews to aid identification of further studies. We identified 540 references and 76 were included in this review.

## 3. Antipsychotic Polypharmacy 

### 3.1. Reasons Behind the Clinical Use of Antipsychotic Polypharmacy

In this article, antipsychotic monotherapy is defined as the use of one antipsychotic for a patient, and antipsychotic polypharmacy is defined as the concurrent use of two or more antipsychotics.

The first reason for using antipsychotic polypharmacy is to treat treatment-resistant schizophrenic patients who did not respond to monotherapy [5]. Clozapine may be used in cases in where antipsychotics treatment failed to produce adequate effects. However, in 1/3–1/5 of the cases, clozapine is ceased due to side effects, or positive symptoms are not remitted even after taking an adequate dose [6]. Polypharmacy is an additional treatment option for such treatment-resistant patients.

Second, antipsychotic polypharmacy may be chosen to induce a rapid drug action for patients exhibiting aggression and violence. Violent behaviors and nervousness may be controlled by using TAP in patients being treated with AAP before the onset of action [7,8]. The fact that a high prevalence of polypharmacy is associated with patient young age, mania, and severe aggression, and that polypharmacy is more commonly used for inpatients or detained patients than for outpatients implies that polypharmacy is used to control acute violent behaviors or nervousness [9]. Among psychiatric inpatients, patients treated with antipsychotic polypharmacy were more frequently diagnosed with schizophrenia and mental retardation as a comorbid illness [10].

Third, polypharmacy is used to reduce the adverse effects resulting from possible overdose of one type of antipsychotic [2,3]. The fact that antipsychotic side effects may be related more to the total dose used in one patient than to the use of polypharmacy suggests that polypharmacy may reduce side effects [11]. Side effects may also be curtailed by using antipsychotics’ affinity for specific receptors. For example, polypharmacy involving aripiprazole, a partial dopamine receptor agonist, may improve plasma prolactin levels [12,13] and metabolic markers [14,15], while reducing side effects, such as somnolence and hypersomnia [16,17].

Fourth, polypharmacy may be chosen to control other symptoms in addition to psychotic symptoms. Quetiapine is one of the most popular antipsychotics used in polypharmacy. Researchers have determined that it has been used to reduce insomnia and anxiety [18]. Finally, two types of antipsychotics may be temporally used in combination when replacing an antipsychotic with another antipsychotic for failing to achieve the desired outcomes with the first drug.

### 3.2. Biological Grounds for Antipsychotic Polypharmacy

The most frequently mentioned evidence for antipsychotic polypharmacy is that it increases dopamine receptor occupancy. All of the approved antipsychotics thus far are dopamine receptor blockers with an efficacy comparable to their affinities for D2 receptors [19,20]. Considering this, the clinical efficacy of antipsychotics may be intensified by increasing D2 receptor blockade in striatum via antipsychotic polypharmacy. A positron emission tomography study reported that more than 60–80% of D2 receptors must be bound for antipsychotics to be effective clinically [21]. Hence, using risperidone with clozapine or quetiapine, which have low affinity at D2 receptors, may increase D2 receptor occupancy [22].

Second, antipsychotic polypharmacy simultaneously blocks or activates several receptors other than the dopamine receptors. For example, although risperidone or ziprasidone monotherapy produces adequate blockade of D2 or 5-HT2 receptors, it does not block adrenergic, muscarinic, and histaminic receptors as effectively; this can be compensated by adding quetiapine or olanzapine to the treatment [11]. As ziprasidone, an agonist of 5-HT1A and D1 receptors, has been confirmed to have antidepressant effects by inhibiting serotonin reuptake, combining it with other antipsychotics would yield additional benefits [23]. Furthermore, aripiprazole, a partial agonist of dopamine, may improve hyperprolactinemia caused by other antipsychotics that block D2 receptors [12].

Third, antipsychotics that are used in combination may mutually influence one another’s metabolism, altering their concentrations in the blood and eventually changing the effects of the medications. Cytochrome P450 enzymes are involved in the metabolism of most antipsychotics [24]. Fluvoxamine or carbamazepine acts as an antagonist or an agonist of the cytochrome P450 enzymes; hence, when taken in combination with other drugs, it can alter their metabolic rates [24,25]. Combining a phenothiazine derivative TAP—thioridazine—may also alter the concentrations of drugs with its antagonistic effects on CYP2D6, an enzyme involved in the metabolism of risperidone and aripiprazole [26]. Most AAPs that are currently in use do not affect CYP450 enzyme activation [24]. However, the metabolic processes of drugs in the human body are not completely predictable, and there have been reports that using clozapine with risperidone increased clozapine concentration [27]. Further, there have been cases in which adding thioridazine, which has antagonistic effects on CYP2D6, to the treatment of patients taking quetiapine, which is metabolized via CYP3A4, activated an unknown metabolic pathway and increased quetiapine clearance by 68% [28]. Hence, such pharmacokinetic changes may serve as the grounds for polypharmacy, given that antipsychotic polypharmacy could potentially alter drug metabolic processes. 

### 3.3. Clinical Grounds for Antipsychotic Polypharmacy

#### 3.3.1. Treatment Resistance in Schizophrenia and Antipsychotics

Only clozapine has demonstrated conclusive favorable evidence in the pharmacological treatment of TRS, which has been consistently reflected in nearly all clinical guidelines [29,30]. Despite great evidence of clozapine in treatment of TRS, clozapine has been underutilized in clinical practice worldwide, and its use has been found to be lower than recommended. Clinician and Patient’s concern over serious side effects such as agranulocytosis or seizures, and feel burdened with continuous laboratory monitoring [31]. However, clinicians should follow their own professional guidelines and prevent their patients from exposing ineffective and dangerous antipsychotic polypharmacy. In a recent review, antipsychotic polypharmacy was found to be superior to monotherapy in symptom reduction, but this superiority was only apparent not in double blind or placebo-controlled (high-quality) trials but in open-label or retrospective (low-quality) trials [32]. Therefore, clozapine monotherapy should be considered first, rather than antipsychotic polypharmacy in the patients with TRS.

Around one third of schizophrenic patients fail to respond to clozapine [6]. These patients are known to have clozapine-resistant or ultra-treatment resistance schizophrenia. For such patients, antipsychotic polypharmacy is a common strategy with a continually growing evidence base [2,3]. In this case, the antipsychotic polypharmacy can be considered in treatment of TRS. Current knowledge about these antipsychotic polypharmacy strategies does not support an evidence based treatment algorithm, but it can be carefully considered based on symptom profile and side effect profile of the patient.

#### 3.3.2. Antipsychotic Polypharmacy with Clozapine

Several randomized controlled trials (RCTs) have been undertaken to examine the effects of combining clozapine with other antipsychotics, namely risperidone [33,34,35,36], sulpiride [37], amisulpride [38,39], and aripiprazole [40,41].

Though RCT evidence is lacking, the clinically most preferred combination of clozapine and risperidone has been suggested as an empirically efficacious treatment regimen [29,30]. Four RCTs that compared the effects of combining clozapine and risperidone showed equivocal results (Table 1). One study reported that risperidone polypharmacy was more efficacious for positive and negative symptoms than clozapine monotherapy [33], and one study showed that polypharmacy was only partially more effective for one of the sub-categories of positive symptoms (disorganized thought) [34]. One study suggested that clozapine monotherapy was more effective [35], and the remaining study found no differences in the efficacy of the two therapies [36]. Although a few other open trial studies have been conducted, their results also conflicted [42,43,44], adding to the ongoing debate on understanding differences in efficacy between monotherapy and polypharmacy.

Table 2 shows RCTs of clozapine, combined with sulpiride, amisulpride, quetiapine, and aripiprazole. An RCT that investigated combining clozapine with sulpiride reported that positive and negative symptoms were significantly improved [37], while another study suggested that combining clozapine with amisulpride was more efficacious than combining it with quetiapine [38]. An RCT of clozapine combination with amisulpride (600 mg/day) showed that psychiatric symptoms were not reduced but the scores of global assessment function, clinical global impression and depression rating scale were improved [39]. Two RCTs studied the efficacy of clozapine and aripiprazole polypharmacy, but the medications failed to improve psychotic symptoms in both studies [40,41]. However, the same study found that the side effects caused by clozapine were controlled and reported that plasma prolactin and triglyceride levels were improved [40,41]. Moreover, while aripiprazole polypharmacy did not lead to symptom improvement, clinical global impression was improved, and body weight, body mass index (BMI), and cholesterol levels decreased [41]. A few other open trial studies have shown that the combination of clozapine and aripiprazole reduced not only metabolic markers, but also some side effects, including somnolence and hypersomnia [16,17].

#### 3.3.3. Antipsychotic Polypharmacy without Clozapine

Thus far, there is a lack of reliable studies verifying the efficacy of antipsychotic polypharmacy without clozapine on patients with TRS [32]. Combinations of olanzapine plus risperidone, quetiapine plus risperidone, and olanzapine plus amisulpride were frequently used, and some studies showed positive outcomes while other studies suggested the need for additional studies to substantiate the efficacy of antipsychotic polypharmacy without clozapine [45]. Most studies on the use of combination of non-clozapine antipsychotics were open label and low-quality trials, and not double blind and high-quality trials, and have shown equivocal results [32]. Some studies have reported that polypharmacy without clozapine was effective for reducing the side effects of antipsychotics or controlling symptoms other than psychotic symptoms such as metabolic adverse effect and hyperprolactinemia [32,45].

### 3.4. Hazards Associated with Antipsychotic Polypharmacy

The first reason underlying the heavy precautions for antipsychotic polypharmacy use is that it eliminates AAP atypicality and may eventually increase adverse effects. Many clinical trials studying the side effects of polypharmacy have associated polypharmacy with increased incidence of side effects, although most have been uncontrolled studies or observational studies [46,47]. Polypharmacy increases the risk for extrapyramidal syndrome and elevation of plasma prolactin concentration [48]. Furthermore, anticholinergics were frequently used for patients who underwent polypharmacy [49]. There have been reports that plasma prolactin concentrations are elevated by polypharmacy not only in the cases involving risperidone [35] or sulpiride [37], both of which have high binding power at dopamine receptors, but also in cases involving drugs that do not have a great effect on plasma prolactin levels, such as quetiapine or olanzapine [50].

Antipsychotic polypharmacy has also been implicated in high patient mortality. Schizophrenic patients show high mortality compared to the general population, primarily owing to poor dietary habits, high substance-related disorder and suicide [51]. Moreover, polypharmacy increases the prevalence of cardiovascular diseases and metabolic syndromes, such as weight gain and diabetes [46], further reducing the survival rate of schizophrenic patients. A 10-year follow-up of schizophrenic patients confirmed that polypharmacy is associated with increased mortality, and a study suggested that increasing the number of prescribed antipsychotics was associated with a higher mortality [52]. However, some large cohort studies have also suggested that antipsychotic polypharmacy was not associated with mortality [53]. Although the existing results on the association between polypharmacy and mortality are conflicting, reports that AAP may increase metabolic disorders are relatively consistent. Thus, polypharmacy should be chosen prudently for patients with cardiovascular disease and metabolic syndrome. Finally, the increased medical costs and financial burden on patients should also be considered when choosing antipsychotic polypharmacy [3].

## 4. Antipsychotics and Metabolic Syndrome

### 4.1. Mechanisms of Weight Gain and Obesity Associated with Atypical Antipsychotics

Recent studies [54,55] have hypothesized that weight gain caused by antipsychotics was a result of interactions between the antipsychotics and a variety of neurotransmitter receptors, including serotonin 5-HT2A and 5-HT2C receptors, histamine H1 receptor, adrenaline α1 and α2 receptors, and muscarinic M3 receptors (Table 3). A recent study investigated the affinities of 17 TAPs and AAPs for 12 neurotransmitter receptors in order to identify the receptors related to weight gain. Affinities for H1 histamine, α1a, 5-HT2c, and 5-HT6 were significantly associated with weight gain, while the remaining eight receptors were not [55]. Among these, 5-HT2C and leptin were speculated to be intimately involved in the mechanism of weight gain induced by antipsychotics. Previous studies have shown that 5-HT2C receptor antagonism and weight gain were closely related, and that a genetic polymorphism at the 5-HT2C receptor promotion site was strongly associated with weight gain in first-onset schizophrenic patients [56,57]. Furthermore, antipsychotic-induced weight gain was also associated with genetic polymorphisms in the leptin gene [58].

Leptin is an anorectic hormone that controls food intake, and it is possible that antipsychotics affect the expression of the leptin gene, inducing weight gain [57]. Clozapine [58] or olanzapine [59] significantly increase plasma leptin concentrations, which is speculated to be due to drug-induced increase of peripheral fat storage that leads to increased leptin release in fat tissues. Some argue that antipsychotics induce increased leptin concentrations by disrupting the leptin pathway in the hypothalamus, thus weakening leptin sensitivity [60].

Weight gain varies depending on the type of antipsychotics and length of use [54]. It most typically occurs within the first 12 weeks of treatment, and being underweight at initial treatment is the greatest risk factor for weight gain [61]. The degree of weight gain and antipsychotics dose are generally presumed to be correlated, and many studies have found that weight increased in a dose-dependent manner [62]. However, the correlation between antipsychotic dosage and degree of weight gain is complex, as weight gain is more influenced by schizophrenia symptom severity than antipsychotic therapy [63].

### 4.2. Mechanisms of Hyperglycemia and Diabetes Mellitus Associated with Atypical Antipsychotics

A retrospective study reported that the prevalence of DM in schizophrenic patients was about 20%, a rate threefold higher than that seen in the general population, and that there was no significant difference in DM incidence between TAP and AAP [64]. The risk for DM varies across the types of drugs involved; olanzapine and clozapine are associated with the highest risks for DM, risperidone, and quetiapine with relatively low risks, while amisulpride, ziprasidone, and aripiprazole, have not been reported to increase the risk for DM [4]. In a 5-year follow-up study of patients using clozapine, 30 out of 82 patients (36.6%) developed DM. Although weight gain contributed to the development of DM in many of the cases, some patients developed DM regardless of weight gain [65]. As shown in this study, DM may develop without a change in body weight, indicating that drug-induced increase of fat is not necessarily a prerequisite for an increase in blood sugar [66].

Although weight gain is not an essential precedent of DM, glucose intolerance is certainly closely linked to weight gain. As antipsychotics induce weight gain to different degrees, it may be assumed that changes in blood sugar control during antipsychotic treatment are entirely secondary to an increase in fat. AAP increases fat within the body via its antagonistic effect on serotonin receptors [56]. An increase in abdominal fat may contribute to hyperglycemia by reducing insulin sensitivity in skeletal muscles [67]. In addition, AAP’s antagonistic action on 5-HT1A receptors among other serotonin receptors seems to reduce the reactivity of pancreatic β cells to blood sugar and impair glucose metabolism [68]. AAP also decreases insulin-sensitive glucose transporters (GLUT) and diminishes GLUT’s ability to migrate to cellular membranes from microsomes [69,70]. Prolonged exposure to high concentrations of sugar and insulin inhibits the translocation of GLUT, thereby diminishing insulin’s ability to transport glucose [71].

In addition to serotonin receptor antagonism and weight gain, changes in the functions of H1 receptors may also contribute to the development of glucose intolerance during the use of AAPs. Abnormalities in H1 receptors disrupt the anorectic effects of leptin, which leads to weight gain and insulin resistance [72]. Antipsychotics associated with the greatest weight gain (e.g., clozapine and olanzapine) show high affinities for H1 receptors, while those associated with little weight gain (e.g., haloperidol and sertindole) show low affinities for H1 receptors [73]. Injecting leptin into the brain of a mouse markedly decreased food intake and body weight, but such effects were not manifested in mice with H1 receptor deficiency [74]. These results imply that an interaction with the H1 receptor-mediating pathway is crucial for leptin to be effective in satiety control and energy consumption. Moreover, some argue that the binding power of antipsychotics to M3 receptors is an important predictor of DM, which is supported by the high expression of M3 receptors in pancreatic beta cells that play a critical role in insulin control [75].

Diabetic ketoacidosis is a serious acute complication of DM with a mortality of about 2–20% [76]. Multiple cases of diabetic ketoacidosis during treatment with clozapine and olanzapine have been reported, and some cases have occurred during treatment with risperidone and quetiapine [77].

### 4.3. Atypical Antipsychotics and Dyslipidemia

Schizophrenic patients have heightened risks for dyslipidemia or hyperlipidemia, and this is related to the schizophrenic patients’ poor dietary and lifestyle habits [78]. Furthermore, dyslipidemia may be exacerbated by particular antipsychotics. In a recent study, clozapine, olanzapine, and quetiapine were reported to increase blood triglyceride and total cholesterol levels, while risperidone and aripiprazole had little or no effect on blood lipids [79]. The specific receptors that play a role in altering lipid metabolism are yet to be identified, but transcription regulatory factors for lipid and carbohydrate metabolism called peroxisome proliferator-activated receptors are thought to be involved [80].

### 4.4. Metabolic Syndrome According to the Type of Atypical Antipsychotic

Olanzapine has been shown to have the greatest effects on weight gain, glucose intolerance, and lipid abnormality as compared to other antipsychotics. In one meta-analysis, the predicted average weight gain after a 10-week treatment was 1.67 kg for risperidone and 4.17 kg for olanzapine, and the proportion of patients who gained weight by more than 7% (cutoff for clinically meaningful weight gain) was higher in the olanzapine group [54]. Multiple studies have consistently reported that olanzapine is associated with glucose intolerance, and olanzapine has also been implicated in increased insulin levels and diabetic ketoacidosis [81,82]. Patients who were treated with perphenazine, quetiapine, risperidone, or ziprasidone showed lower glycosylated hemoglobin and low glucose levels when compared to those who were treated with olanzapine [83]. Further, olanzapine increased the accumulation of body fat, which in turn was associated with dyslipidemia, such as increased triglyceride [81].

The prevalence of metabolic syndrome was significantly higher among patients who took clozapine than among the general population. However, it was difficult to compare this to patients taking other antipsychotics due to the lack of data revealing the prevalence of morbidities in association with particular antipsychotics [84]. In a study on 242 patients taking clozapine who developed DM for the first time, most patients were found to have developed DM within the first six months of treatment [85]. However, patients without DM risk factors were diagnosed with DM between 10–27 months after the initiation of antipsychotic regimens. Thus, the presence of DM risk factors was presumed important to determine the specific onset of DM in relation to AAP.

Treatment with clozapine and olanzapine itself may induce insulin resistance in schizophrenic patients regardless of weight gain. In fact, some patients have shown signs of insulin resistance even without gaining weight [86]. Rapid onset of DM and loss of hyperglycemia after cessation of medications are particularly more prominent in cases involving olanzapine and clozapine. Meanwhile, a study that examined the effects of various antipsychotics on insulin secretion reported that clozapine stimulated insulin release by pancreatic beta cells while olanzapine did not significantly increase insulin secretion [87].

There was a report that 10-week administration of risperidone led to an average of 1.67 kg of weight gain [88]. However, after an 8-week treatment with risperidone, clozapine, olanzapine, or sulpiride, risperidone was the only drug that did not result in significant increases in BMI and waist hip ratio (WHR) [89]. It has been reported that risperidone tends to lower cholesterol and triglyceride levels [90]. In conclusion, weight gain may also be a problem with the use of risperidone, but compared to olanzapine and clozapine, risperidone seems to have less effect on lipid metabolism.

About 50% of the patients who took quetiapine gained weight by more than 7%, while more than 80% in the olanzapine group and 58% in the risperidone group gained weight by more than 7% [91]. Quetiapine induced a smaller weight and BMI gain in female patients than in male patients [91]. Recent studies have associated clozapine, olanzapine, and quetiapine, with increased total cholesterol and triglyceride levels, but quetiapine has been found to have weaker associations when compared to clozapine and olanzapine [83].

Recent studies assert that aripiprazole is safer than other drugs in terms of metabolic syndrome [92]. In addition, as compared to other AAPs, aripiprazole has been suggested to have benefits in changes in metabolic parameters [15]. A retrospective study found that switching to aripiprazole from another AAP led to a significant decrease in total cholesterol and body weight [15]. When considering the above studies, aripiprazole may be preferentially chosen for patients carrying risk factors for metabolic disorders. Further, replacing the treatment regimen with aripiprazole may be an option for patients who have developed metabolic side effects from AAPs. However, one study suggested that merely replacing an original drug with aripiprazole did not improve metabolic abnormalities in patients who had already developed metabolic abnormalities due to AAPs, such as weight gain [93,94]. Thus, instead of resorting to a monotherapy strategy of changing an original drug to aripiprazole, a combined therapy that includes exercise, limited calorie intake, and improvement of dietary habits should be performed to acquire maximum benefits. During aripiprazole treatment, an oral glucose tolerance test showed reductions in glucose and insulin levels. After a three-month treatment with aripiprazole, total cholesterol, triglyceride, and low-density lipoprotein cholesterol levels dropped, but high-density lipoprotein cholesterol levels remained unchanged [94]. Ziprasidone seems to have no effect on weight gain but lowers cholesterol and triglycerides without altering glucose utilization [95].

## 5. Further Considerations

### Atypical Antipsychotics and Suicidality

Despite the emergence of AAP, suicide rates for schizophrenic patients are still high. Another important therapeutic purpose in using AAP in schizophrenic patients is to prevent suicide. Patient’s insight, which is a factor that protects from relapses and recurrences, has been considered a major factor for suicide in schizophrenia [96]. Gradual gains in insight brought by successful AAP treatment may decrease suicidal risk [96,97]. However, it is unclear whether most atypical antipsychotics are directly effective in preventing suicide [98]. Clozapine is the only AAP that gathered evidence as an effective treatment for reducing suicide risk in patients with schizophrenia [99]. It has been reported that clozapine has an antidepressant properties on its own [100]. Further studies will be required to determine whether there is a direct pharmacologic link between antidepressant effect and reduced suicide rates in schizophrenia for clozapine, or whether other pharmacologic factors might be involved. Additional researches are also required to confirm if there are substantial reductions in suicide rates when newer AAPs are given to patients with schizophrenia. The AAP are actually a heterogeneous group of agents, and they may show different antidepressant or anti-suicide properties in ways that have by no means yet been fully investigated, and the related mechanisms remain to be definitively explicated.

## 6. Conclusions and Future Directions

Despite the passive attitudes maintained in clinical treatment guidelines, antipsychotics polypharmacy is used quite frequently in the clinical setting. In many cases, clinicians choose polypharmacy to control the symptoms of treatment-resistant schizophrenic patients or the side effects that occur as a result of using antipsychotic monotherapy. Theoretical grounds for polypharmacy include the fact that polypharmacy can alter the activation of various receptors, including D2 receptors, and that it can induce pharmacokinetic changes. Some RCTs have reported that when combining clozapine with risperidone, sulpiride, or amisulpride, was effective in clozapine-resistant patients, and that aripiprazole polypharmacy may reduce the incidence of hyperprolactinemia or metabolic side effects. However, there are also reports that polypharmacy increases the prevalence of dose-dependent side effects and metabolic side effects, and that it is related to increased mortality. Thus, patients’ past medication history should be meticulously reviewed to determine the need to use antipsychotic polypharmacy, and if needed, clear goals should be set to use antipsychotic polypharmacy appropriately. 

AAP is safer than TAP in terms of side effects, but it can cause metabolic syndrome, including weight gain, glucose intolerance, and dyslipidemia. Antipsychotics are believed to have great effects on weight gain according to their binding to serotoninergic (5-HT2C, 5-HT2A, and 5-HT1A) and histaminergic (H1 and H2) receptors. Olanzapine and clozapine were associated with higher prevalence of weight gain and glucose intolerance as compared to other AAPs, while aripiprazole and ziprasidone have been reported to be associated with little or no metabolic syndrome. Predicting and preventing the development of metabolic abnormalities in patients using AAP are important. Careful monitoring of serum glucose, cholesterol level, and weight gain are the most important things to prevent AAP related metabolic syndrome. Future studies should develop specific treatment strategies to control metabolic abnormalities and identify the mechanisms of metabolic side effects and related receptors.

## Figures and Tables

**Table 1:** Summary of randomized controlled studies of clozapine combined with risperidone.

Study	Patients	Dose and Duration	Results	Adverse Effect
Josiassen et al. [33]	SPR no or partial response to CZP; *n* = 40	Double-blind; 12 weeks; CZP (mean 529 mg/d) + RIS up to 6 mg/d (*n* = 20) vs. CZP (mean 403 mg/d) + PLC (*n* = 20)	Significantly greater reduction with CZP + RIS than CZP + PLC on BPRS	
Freudenreich et al. [34]	SPR partial response to CZP; *n* = 24	Double-blind; 6 weeks; CZP + RIS 4 mg/d (*n* = 12) vs. CZP + PLC (*n* = 12)	No significant differences in PANSS total score, although significant improvement in subscale “thought disorganization” under RIS	
Anil Yagcioglu et al. [35]	SPR partial response to CZP; *n* = 30	Double-blind; 6 weeks; CZP (mean 516 mg/d) + RIS up to 6 mg/d (*n* = 16) vs. CZP (mean 414 mg/d) + PLC (*n* = 14)	Significant improvement in PANSS positive subscale and single cognitive functions in the PLC group	Under RIS significantly more sedation and prolactin increase
Honer et al. [36]	SPR poor response to CZP; *n* = 68	Double-blind; 8 weeks; CZP (mean 494 mg/d) + RIS up to 3 mg/d (*n* = 34) vs. CZP (mean 487 mg/d) + PLC (*n* = 34)	No differences in PANSS between the groups, significant slight improvement in verbal working memory under PLC	Significant slight increase in fasting glucose level in the RIS group

SPR: schizophrenia, CZP: clozapine, RIS: risperidone, PLC: placebo, BPRS: Brief Psychiatric Rating Scale, PANSS: Positive and Negative Syndrome Scale.

**Table 2:** Summary of randomized controlled studies of clozapine combined with sulpiride, amisulpride, quetiapine, and aripiprazole.

Study	Patients	Dose and Duration	Results	Adverse Effect
Shiloh et al. [37]	SPR partial response to CZP; *n* = 28	Double-blind; 10 weeks; CZP (mean 425 mg/d) + PLC (*n* = 12) vs. CZP (mean 425 mg/d) + SUL 600 mg/d (*n* = 16)	Significant improvement in BPRS total score, SAPS, SANS under the combination, together	Significant prolactin increase, worsening of the pre-existing tardive dyskinesia in one patient
Genç et al. [38]	SPR partial response to CZP; *n* = 56	Single-blind; 8 weeks; CZP + AMI up to 800 mg/d (*n* = 28) vs. CZP + QUE up to 900 mg/d (*n* = 28)	Significant improvement in BPRS, SAPS, SANS, CGI under combination with AMI	
Assion et al. [39]	SPR partial response to CZP; *n* = 16	Double-blind; 6 weeks; CZP + AMI 400 mg/d (*n* = 7) vs. CZP + AMI 600 mg/d (*n* = 6) vs. CZP + PLC (*n* = 3)	Significant improvement in GAF, CGI and MADRS under combination with AMI 600 mg, no reduction in BPRS total score	Tremor, bradykinesia, akathisia and elevated prolactin levels were recorded
Chang et al. [40]	SPR no or partial response to CZP; *n* = 62	double-blind; 8 weeks; CZP (mean 290.6 mg/d) + PLC (*n* = 32) vs. CZP (mean 304.3 mg/d) + ARP (*n* = 29)	No significant differences in BPRS total score, significant improvement BPRS, negative symptom subscale, SANS total score; prolactin and triglyceride levels were significantly lower in the ARP	
Fleischhacker et al. [41]	SPR Partial response to CZP, weight gain ≥ 2.5 kg; *n* = 207	double-blind;16 weeks; CLZ (mean 363 mg/d) + PLC (*n* = 99) vs. CZP (384 mg/d) + ARP (*n* = 108)	No significant differences in PANSS significant weight loss, BMI, waist circumference, LDL cholesterol reduction	

SPR: schizophrenia, CZP: clozapine, SUL: sulpiride, AMI: amisulpride, ARP: aripiprazole, QUE: quetiapine, PLC: placebo, BPRS: Brief Psychiatric Rating Scale, PANSS: Positive and Negative Syndrome Scale, SAPS: Scale for the Assessment of Positive Symptoms, SANS: Scale for the Assessment of Negative Symptoms, CGI: Clinical Global Impression, GAF: Global Assessment Function, MADRS: Montgomery-Asberg Depression Rating Scale, BMI: body mass index, LDL: low density lipoprotein.

**Table 3:** Receptors related to metabolic abnormalities.

Metabolic Syndrome	Receptor Activity						
	H1	H3	5-HT1A	5HT-2C	M3	D2	PPARS
Weight gain	(−)	(+)	(−)	(−)		(−)	
Glucose dysregulation	(−)			(−)	(−)		
Dyslipidemia							(−)

(+): Agonism, (−): Antagonism, PPARs: Peroxisome proliferator-activated receptors.

## References

[1] Orsolini L., Tomasetti C., Valchera A., Vecchiotti R., Matarazzo I., Vellante F., Iasevoli F., Buonaguro E.F., Fornaro M., Fiengo A.L. (2016). An update of safety of clinically used atypical antipsychotics. Expert Opin. Drug Saf..

[2] Mojtabai R., Olfson M. (2010). National trends in psychotropic medication polypharmacy in office-based psychiatry. Arch. Gen. Psychiatry.

[3] Biancosino B., Barbui C., Marmai L., Dona S., Grassi L. (2005). Determinants of antipsychotic polypharmacy in psychiatric inpatients: A prospective study. Int. Clin. Psychopharmacol..

[4] Reynolds G.P., Kirk S.L. (2010). Metabolic side effects of antipsychotic drug treatment-pharmacological mechanisms. Pharmacol. Ther..

[5] Wheeler A. (2009). Explicit versus implicit review to explore combination antipsychotic prescribing. J. Eval. Clin. Pract..

[6] Conley R.R., Kelly D.L. (2001). Management of treatment resistance in schizophrenia. Biol. Psychiatry.

[7] Bitter I., Czobor P., Dossenbach M., Volavka J. (2005). Effectiveness of clozapine, olanzapine, quetiapine, risperidone, and haloperidol monotherapy in reducing hostile and aggressive behavior in outpatients treated for schizophrenia: A prospective naturalistic study (IC-SOHO). Eur. Psychiatry.

[8] Volavka J., Czobor P., Citrome L., Van Dorn R.A. (2014). Effectiveness of antipsychotic drugs against hostility in patients with schizophrenia in the Clinical Antipsychotic Trials of Intervention Effectiveness (CATIE) study. CNS Spectr..

[9] Xiang Y.T., Wang C.Y., Si T.M., Lee E.H., He Y.L., Ungvari G.S., Chiu H.F., Yang S.Y., Chong M.Y., Tan C.H. (2012). Antipsychotic polypharmacy in inpatients with schizophrenia in Asia (2001–2009). Pharmacopsychiatry.

[10] Iasevoli F., Buonaguro E.F., Marconi M., Di Giovambattista E., Rapagnani M.P., De Berardis D., Martinotti G., Mazza M., Balletta R., Serroni N. (2014). Efficacy and clinical determinants of antipsychotic polypharmacy in psychotic patients experiencing an acute relapse and admitted to hospital stay: Results from a cross-sectional and a subsequent longitudinal pilot study. ISRN Pharmacol..

[11] Freudenreich O., Goff D.C. (2002). Antipsychotic combination therapy in schizophrenia. A review of efficacy and risks of current combinations. Acta Psychiatr. Scand..

[12] Li X., Tang Y., Wang C. (2013). Adjunctive aripiprazole versus placebo for antipsychotic-induced hyperprolactinemia: Meta-analysis of randomized controlled trials. PLoS ONE.

[13] Shim J.C., Shin J.G., Kelly D.L., Jung D.U., Seo Y.S., Liu K.H., Shon J.H., Conley R.R. (2007). Adjunctive treatment with a dopamine partial agonist, aripiprazole, for antipsychotic-induced hyperprolactinemia: A placebo-controlled trial. Am. J. Psychiatry.

[14] Stroup T.S., McEvoy J.P., Ring K.D., Hamer R.H., LaVange L.M., Swartz M.S., Rosenheck R.A., Perkins D.O., Nussbaum A.M., Lieberman J.A. (2011). A randomized trial examining the effectiveness of switching from olanzapine, quetiapine, or risperidone to aripiprazole to reduce metabolic risk: Comparison of antipsychotics for metabolic problems (CAMP). Am. J. Psychiatry.

[15] Chen Y., Bobo W.V., Watts K., Jayathilake K., Tang T., Meltzer H.Y. (2012). Comparative effectiveness of switching antipsychotic drug treatment to aripiprazole or ziprasidone for improving metabolic profile and atherogenic dyslipidemia: A 12-month, prospective, open-label study. J. Psychopharmacol..

[16] Karunakaran K., Tungaraza T.E., Harborne G.C. (2007). Is clozapine-aripiprazole combination a useful regime in the management of treatment-resistant schizophrenia?. J. Psychopharmacol..

[17] Rocha F.L., Hara C. (2006). Benefits of combining aripiprazole to clozapine: Three case reports. Prog. Neuropsychopharmacol. Biol. Psychiatry.

[18] Correll C.U., Shaikh L., Gallego J.A., Nachbar J., Olshanskiy V., Kishimoto T., Kane J.M. (2011). Antipsychotic polypharmacy: A survey study of prescriber attitudes, knowledge and behavior. Schizophr. Res..

[19] Lako I.M., van den Heuvel E.R., Knegtering H., Bruggeman R., Taxis K. (2013). Estimating dopamine D(2) receptor occupancy for doses of 8 antipsychotics: A meta-analysis. J. Clin. Psychopharmacol..

[20] Yilmaz Z., Zai C.C., Hwang R., Mann S., Arenovich T., Remington G., Daskalakis Z.J. (2012). Antipsychotics, dopamine D(2) receptor occupancy and clinical improvement in schizophrenia: A meta-analysis. Schizophr. Res..

[21] Nordstrom A.L., Farde L., Wiesel F.A., Forslund K., Pauli S., Halldin C., Uppfeldt G. (1993). Central D2-dopamine receptor occupancy in relation to antipsychotic drug effects: A double-blind PET study of schizophrenic patients. Biol. Psychiatry.

[22] Uchida H., Takeuchi H., Graff-Guerrero A., Suzuki T., Watanabe K., Mamo D.C. (2011). Predicting dopamine D(2) receptor occupancy from plasma levels of antipsychotic drugs: A systematic review and pooled analysis. J. Clin. Psychopharmacol..

[23] Nemeroff C.B., Lieberman J.A., Weiden P.J., Harvey P.D., Newcomer J.W., Schatzberg A.F., Kilts C.D., Daniel D.G. (2005). From clinical research to clinical practice: A 4-year review of ziprasidone. CNS Spectr..

[24] Spina E., de Leon J. (2007). Metabolic drug interactions with newer antipsychotics: A comparative review. Basic Clin. Pharmacol. Toxicol..

[25] Hiemke C., Weigmann H., Hartter S., Dahmen N., Wetzel H., Muller H. (1994). Elevated levels of clozapine in serum after addition of fluvoxamine. J. Clin. Psychopharmacol..

[26] Mulsant B.H., Foglia J.P., Sweet R.A., Rosen J., Lo K.H., Pollock B.G. (1997). The effects of perphenazine on the concentration of nortriptyline and its hydroxymetabolites in older patients. J. Clin. Psychopharmacol..

[27] Tyson S.C., Devane C.L., Risch S.C. (1995). Pharmacokinetic interaction between risperidone and clozapine. Am. J. Psychiatry.

[28] Potkin S.G., Thyrum P.T., Alva G., Bera R., Yeh C., Arvanitis L.A. (2002). The safety and pharmacokinetics of quetiapine when coadministered with haloperidol, risperidone, or thioridazine. J. Clin. Psychopharmacol..

[29] Hasan A., Falkai P., Wobrock T., Lieberman J., Glenthoj B., Gattaz W.F., Thibaut F., Moller H.J. (2017). World Federation of Societies of Biological Psychiatry (WFSBP) guidelines for biological treatment of schizophrenia—A short version for primary care. Int. J. Psychiatry Clin. Pract..

[30] Galletly C., Castle D., Dark F., Humberstone V., Jablensky A., Killackey E., Kulkarni J., McGorry P., Nielssen O., Tran N. (2016). Royal Australian and New Zealand College of Psychiatrists clinical practice guidelines for the management of schizophrenia and related disorders. Aust. N. Z. J. Psychiatry.

[31] Bogers J.P., Schulte P.F., Van Dijk D., Bakker B., Cohen D. (2016). Clozapine Underutilization in the Treatment of Schizophrenia: How Can Clozapine Prescription Rates Be Improved?. J. Clin. Psychopharmacol..

[32] Galling B., Roldan A., Hagi K., Rietschel L., Walyzada F., Zheng W., Cao X.L., Xiang Y.T., Zink M., Kane J.M. (2017). Antipsychotic augmentation vs. monotherapy in schizophrenia: Systematic review, meta-analysis and meta-regression analysis. World Psychiatry.

[33] Josiassen R.C., Joseph A., Kohegyi E., Stokes S., Dadvand M., Paing W.W., Shaughnessy R.A. (2005). Clozapine augmented with risperidone in the treatment of schizophrenia: A randomized, double-blind, placebo-controlled trial. Am. J. Psychiatry.

[34] Freudenreich O., Henderson D.C., Walsh J.P., Culhane M.A., Goff D.C. (2007). Risperidone augmentation for schizophrenia partially responsive to clozapine: A double-blind, placebo-controlled trial. Schizophr. Res..

[35] Anil Yagcioglu A.E., Kivircik Akdede B.B., Turgut T.I., Tumuklu M., Yazici M.K., Alptekin K., Ertugrul A., Jayathilake K., Gogus A., Tunca Z. (2005). A double-blind controlled study of adjunctive treatment with risperidone in schizophrenic patients partially responsive to clozapine: Efficacy and safety. J. Clin. Psychiatry.

[36] Honer W.G., Thornton A.E., Chen E.Y., Chan R.C., Wong J.O., Bergmann A., Falkai P., Pomarol-Clotet E., McKenna P.J., Stip E. (2006). Clozapine alone versus clozapine and risperidone with refractory schizophrenia. N. Engl. J. Med..

[37] Shiloh R., Zemishlany Z., Aizenberg D., Radwan M., Schwartz B., Dorfman-Etrog P., Modai I., Khaikin M., Weizman A. (1997). Sulpiride augmentation in people with schizophrenia partially responsive to clozapine. A double-blind, placebo-controlled study. Br. J. Psychiatry.

[38] Genc Y., Taner E., Candansayar S. (2007). Comparison of clozapine-amisulpride and clozapine-quetiapine combinations for patients with schizophrenia who are partially responsive to clozapine: A single-blind randomized study. Adv. Ther..

[39] Assion H.J., Reinbold H., Lemanski S., Basilowski M., Juckel G. (2008). Amisulpride augmentation in patients with schizophrenia partially responsive or unresponsive to clozapine. A randomized, double-blind, placebo-controlled trial. Pharmacopsychiatry.

[40] Chang J.S., Ahn Y.M., Park H.J., Lee K.Y., Kim S.H., Kang U.G., Kim Y.S. (2008). Aripiprazole augmentation in clozapine-treated patients with refractory schizophrenia: An 8-week, randomized, double-blind, placebo-controlled trial. J. Clin. Psychiatry.

[41] Fleischhacker W.W., Heikkinen M.E., Olie J.P., Landsberg W., Dewaele P., McQuade R.D., Loze J.Y., Hennicken D., Kerselaers W. (2010). Effects of adjunctive treatment with aripiprazole on body weight and clinical efficacy in schizophrenia patients treated with clozapine: A randomized, double-blind, placebo-controlled trial. Int. J. Neuropsychopharmacol..

[42] Henderson D.C., Goff D.C. (1996). Risperidone as an adjunct to clozapine therapy in chronic schizophrenics. J. Clin. Psychiatry.

[43] Taylor C.G., Flynn S.W., Altman S., Ehmann T., MacEwan G.W., Honer W.G. (2001). An open trial of risperidone augmentation of partial response to clozapine. Schizophr. Res..

[44] De Groot I.W., Heck A.H., van Harten P.N. (2001). Addition of risperidone to clozapine therapy in chronically psychotic inpatients. J. Clin. Psychiatry.

[45] Canadian Agency for Drugs and Technologies in Health (2012). Combination and high-dose atypical antipsychotic therapy in patients with schizophrenia: Systematic review. CADTH Technol. Overv..

[46] Misawa F., Shimizu K., Fujii Y., Miyata R., Koshiishi F., Kobayashi M., Shida H., Oguchi Y., Okumura Y., Ito H. (2011). Is antipsychotic polypharmacy associated with metabolic syndrome even after adjustment for lifestyle effects? A cross-sectional study. BMC Psychiatry.

[47] Ray W.A., Chung C.P., Murray K.T., Hall K., Stein C.M. (2009). Atypical antipsychotic drugs and the risk of sudden cardiac death. N. Engl. J. Med..

[48] Gallego J.A., Nielsen J., De Hert M., Kane J.M., Correll C.U. (2012). Safety and tolerability of antipsychotic polypharmacy. Expert Opin. Drug Saf..

[49] Chakos M.H., Glick I.D., Miller A.L., Hamner M.B., Miller D.D., Patel J.K., Tapp A., Keefe R.S., Rosenheck R.A. (2006). Baseline use of concomitant psychotropic medications to treat schizophrenia in the CATIE trial. Psychiatr. Serv..

[50] Montgomery J., Winterbottom E., Jessani M., Kohegyi E., Fulmer J., Seamonds B., Josiassen R.C. (2004). Prevalence of hyperprolactinemia in schizophrenia: Association with typical and atypical antipsychotic treatment. J. Clin. Psychiatry.

[51] De Hert M., Cohen D., Bobes J., Cetkovich-Bakmas M., Leucht S., Ndetei D.M., Newcomer J.W., Uwakwe R., Asai I., Moller H.J. (2011). Physical illness in patients with severe mental disorders. II. Barriers to care, monitoring and treatment guidelines, plus recommendations at the system and individual level. World Psychiatry.

[52] Torniainen M., Mittendorfer-Rutz E., Tanskanen A., Bjorkenstam C., Suvisaari J., Alexanderson K., Tiihonen J. (2015). Antipsychotic treatment and mortality in schizophrenia. Schizophr. Bull..

[53] Baandrup L., Sorensen J., Lublin H., Nordentoft M., Glenthoj B. (2012). Association of antipsychotic polypharmacy with health service cost: A register-based cost analysis. Eur. J. Health Econ..

[54] Bak M., Fransen A., Janssen J., van Os J., Drukker M. (2014). Almost all antipsychotics result in weight gain: A meta-analysis. PLoS ONE.

[55] Montastruc F., Palmaro A., Bagheri H., Schmitt L., Montastruc J.L., Lapeyre-Mestre M. (2015). Role of serotonin 5-HT2C and histamine H1 receptors in antipsychotic-induced diabetes: A pharmacoepidemiological-pharmacodynamic study in VigiBase. Eur. Neuropsychopharmacol..

[56] Reynolds G.P., Hill M.J., Kirk S.L. (2006). The 5-HT2C receptor and antipsychoticinduced weight gain—Mechanisms and genetics. J. Psychopharmacol..

[57] Panariello F., Polsinelli G., Borlido C., Monda M., De Luca V. (2012). The role of leptin in antipsychotic-induced weight gain: Genetic and non-genetic factors. J. Obes..

[58] Kang S.H., Lee J.I., Han H.R., Soh M., Hong J.P. (2014). Polymorphisms of the leptin and HTR2C genes and clozapine-induced weight change and baseline BMI in patients with chronic schizophrenia. Psychiatr. Genet..

[59] Tsuneyama N., Suzuki Y., Sawamura K., Sugai T., Fukui N., Watanabe J., Ono S., Saito M., Someya T. (2016). Effect of Serum Leptin on Weight Gain Induced by Olanzapine in Female Patients with Schizophrenia. PLoS ONE.

[60] Cortes B., Becker J., Mories Alvarez M.T., Marcos A.I., Molina V. (2014). Contribution of baseline body mass index and leptin serum level to the prediction of early weight gain with atypical antipsychotics in schizophrenia. Psychiatry Clin. Neurosci..

[61] Wetterling T. (2001). Bodyweight gain with atypical antipsychotics. A comparative review. Drug Saf..

[62] Simon V., van Winkel R., De Hert M. (2009). Are weight gain and metabolic side effects of atypical antipsychotics dose dependent? A literature review. J. Clin. Psychiatry.

[63] Kalucy R.S. (1980). Drug-induced weight gain. Drugs.

[64] Buse J.B., Cavazzoni P., Hornbuckle K., Hutchins D., Breier A., Jovanovic L. (2003). A retrospective cohort study of diabetes mellitus and antipsychotic treatment in the United States. J. Clin. Epidemiol..

[65] Henderson D.C., Cagliero E., Gray C., Nasrallah R.A., Hayden D.L., Schoenfeld D.A., Goff D.C. (2000). Clozapine, diabetes mellitus, weight gain, and lipid abnormalities: A five-year naturalistic study. Am. J. Psychiatry.

[66] Wysokinski A. (2014). Blood levels of glucose and insulin and insulin resistance in patients with schizophrenia on clozapine monotherapy. Diabetes Metab. Syndr..

[67] Muller M.J., Lagerpusch M., Enderle J., Schautz B., Heller M., Bosy-Westphal A. (2012). Beyond the body mass index: Tracking body composition in the pathogenesis of obesity and the metabolic syndrome. Obes. Rev..

[68] Kelly A.C., Sheitman B.B., Hamer R.M., Rhyne D.C., Reed R.M., Graham K.A., Rau S.W., Gilmore J.H., Perkins D.O., Peebles S.S. (2014). A naturalistic comparison of the long-term metabolic adverse effects of clozapine versus other antipsychotics for patients with psychotic illnesses. J. Clin. Psychopharmacol..

[69] Taniguchi A., Nakai Y., Fukushima M., Kawamura H., Imura H., Nagata I., Tokuyama K. (1992). Pathogenic factors responsible for glucose intolerance in patients with NIDDM. Diabetes.

[70] Taniguchi A., Nakai Y., Fukushima M., Imura H., Kawamura H., Nagata I., Florant G.L., Tokuyama K. (1994). Insulin sensitivity, insulin secretion, and glucose effectiveness in subjects with impaired glucose tolerance: A minimal model analysis. Metabolism.

[71] Kopelman P.G., Albon L. (1997). Obesity, non-insulin-dependent diabetes mellitus and the metabolic syndrome. Br. Med. Bull..

[72] He M., Deng C., Huang X.F. (2013). The role of hypothalamic H1 receptor antagonism in antipsychotic-induced weight gain. CNS Drugs.

[73] Han M., Deng C., Burne T.H., Newell K.A., Huang X.F. (2008). Short- and long-term effects of antipsychotic drug treatment on weight gain and H1 receptor expression. Psychoneuroendocrinology.

[74] Schneider E.H., Neumann D., Seifert R. (2014). Modulation of behavior by the histaminergic system: Lessons from H(1)R-and H(2)R-deficient mice. Neurosci. Biobehav. Rev..

[75] Silvestre J.S., Prous J. (2005). Research on adverse drug events. I. Muscarinic M3 receptor binding affinity could predict the risk of antipsychotics to induce type 2 diabetes. Methods Find. Exp. Clin. Pharmacol..

[76] Malone M.L., Gennis V., Goodwin J.S. (1992). Characteristics of diabetic ketoacidosis in older versus younger adults. J. Am. Geriatr. Soc..

[77] Hepburn K., Brzozowska M.M. (2016). Diabetic ketoacidosis and severe hypertriglyceridaemia as a consequence of an atypical antipsychotic agent. BMJ Case Rep..

[78] Casey D.E. (2004). Dyslipidemia and atypical antipsychotic drugs. J. Clin. Psychiatry.

[79] Yogaratnam J., Biswas N., Vadivel R., Jacob R. (2013). Metabolic complications of schizophrenia and antipsychotic medications—An updated review. East. Asian Arch. Psychiatry.

[80] Arulmozhi D.K., Dwyer D.S., Bodhankar S.L. (2006). Antipsychotic induced metabolic abnormalities: An interaction study with various PPAR modulators in mice. Life Sci..

[81] Iwaku K., Otuka F., Taniyama M. (2017). Acute-Onset Type 1 Diabetes that Developed During the Administration of Olanzapine. Intern. Med..

[82] Kohen I., Gampel M., Reddy L., Manu P. (2008). Rapidly developing hyperglycemia during treatment with olanzapine. Ann. Pharmacother..

[83] Nasrallah H.A. (2006). Metabolic findings from the CATIE trial and their relation to tolerability. CNS Spectr..

[84] Hyde N., Dodd S., Venugopal K., Purdie C., Berk M., O’Neil A. (2015). Prevalence of cardiovascular and metabolic events in patients prescribed clozapine: A retrospective observational, clinical cohort study. Curr. Drug Saf..

[85] Koller E., Schneider B., Bennett K., Dubitsky G. (2001). Clozapine-associated diabetes. Am. J. Med..

[86] Chaggar P.S., Shaw S.M., Williams S.G. (2011). Effect of antipsychotic medications on glucose and lipid levels. J. Clin. Pharmacol..

[87] Melkersson K., Jansson E. (2007). Effects of the atypical antipsychotic clozapine on insulin release in vitro. Neuro Endocrinol. Lett..

[88] Conley R.R., Mahmoud R. (2001). A randomized double-blind study of risperidone and olanzapine in the treatment of schizophrenia or schizoaffective disorder. Am. J. Psychiatry.

[89] Wu R.R., Zhao J.P., Liu Z.N., Zhai J.G., Guo X.F., Guo W.B., Tang J.S. (2006). Effects of typical and atypical antipsychotics on glucose-insulin homeostasis and lipid metabolism in first-episode schizophrenia. Psychopharmacology (Berl.).

[90] Strous R.D., Kupchik M., Roitman S., Schwartz S., Gonen N., Mester R., Weizman A., Spivak B. (2006). Comparison between risperidone, olanzapine, and clozapine in the management of chronic schizophrenia: A naturalistic prospective 12-week observational study. Hum. Psychopharmacol..

[91] McEvoy J.P., Lieberman J.A., Perkins D.O., Hamer R.M., Gu H., Lazarus A., Sweitzer D., Olexy C., Weiden P., Strakowski S.D. (2007). Efficacy and tolerability of olanzapine, quetiapine, and risperidone in the treatment of early psychosis: A randomized, double-blind 52-week comparison. Am. J. Psychiatry.

[92] Moteshafi H., Stip E. (2012). Comparing tolerability profile of quetiapine, risperidone, aripiprazole and ziprasidone in schizophrenia and affective disorders: A meta-analysis. Expert Opin. Drug Saf..

[93] Kim S.H., Ivanova O., Abbasi F.A., Lamendola C.A., Reaven G.M., Glick I.D. (2007). Metabolic impact of switching antipsychotic therapy to aripiprazole after weight gain: A pilot study. J. Clin. Psychopharmacol..

[94] De Hert M., Hanssens L., van Winkel R., Wampers M., Van Eyck D., Scheen A., Peuskens J. (2007). A case series: Evaluation of the metabolic safety of aripiprazole. Schizophr. Bull..

[95] Mandrioli R., Protti M., Mercolini L. (2015). Evaluation of the pharmacokinetics, safety and clinical efficacy of ziprasidone for the treatment of schizophrenia and bipolar disorder. Expert Opin. Drug Metab. Toxicol..

[96] Pompili M., Ruberto A., Kotzalidis G.D., Girardi P., Tatarelli R. (2004). Suicide and awareness of illness in schizophrenia: An overview. Bull. Menninger Clin..

[97] Pompili M., Baldessarini R.J., Forte A., Erbuto D., Serafini G., Fiorillo A., Amore M., Girardi P. (2016). Do Atypical Antipsychotics Have Antisuicidal Effects? A Hypothesis-Generating Overview. Int. J. Mol. Sci..

[98] Pompili M., Orsolini L., Lamis D.A., Goldsmith D.R., Nardella A., Falcone G., Corigliano V., Luciano M., Fiorillo A. (2017). Suicide Prevention in Schizophrenia: Do Long-Acting Injectable Antipsychotics (LAIs) have a Role?. CNS Neurol. Disord. Drug Targets.

[99] Reid W.H., Mason M., Hogan T. (1998). Suicide prevention effects associated with clozapine therapy in schizophrenia and schizoaffective disorder. Psychiatr. Serv..

[100] Ranjan R., Meltzer H.Y. (1996). Acute and long-term effectiveness of clozapine in treatment-resistant psychotic depression. Biol. Psychiatry.

